# The Shannon–McMillan Theorem Proves Convergence to Equiprobability of Boltzmann’s Microstates

**DOI:** 10.3390/e23070899

**Published:** 2021-07-15

**Authors:** Arnaldo Spalvieri

**Affiliations:** Dipartimento di Elettronica, Informazione e Bioingegneria, Politecnico di Milano, 20133 Milan, Italy; arnaldo.spalvieri@polimi.it

**Keywords:** Boltzmann–Planck entropy formula, Shannon–McMillan theorem, equiprobability of microstates

## Abstract

This paper shows that, for a large number of particles and for distinguishable and non-interacting identical particles, convergence to equiprobability of the *W* microstates of the famous Boltzmann–Planck entropy formula *S* = *k* log(*W*) is proved by the Shannon–McMillan theorem, a cornerstone of information theory. This result further strengthens the link between information theory and statistical mechanics.

## 1. Introduction

Entropy plays a key role in communication and information theory and in thermodynamics. Starting from the concept of entropy, many authors in the past built bridges between information and communication theory and thermodynamics, such that all the textbooks of statistical mechanics and thermal physics have chapters or sections where the basics of information theory are introduced. A recent survey about the links between entropy in communications and in thermodynamics is [1]. Making a comprehensive review of the bibliography on this topic is out of the scope of the present paper. We limit ourselves to mentioning the pioneering work of Jaynes [2] and the attention that has been attracted in the past by the connections between information theoretic inequalities and the irreversibility of certain thermodynamical processes; see [3], Chapter 4 of [4] and [5].

The aim of this paper is to put the information-theoretic concept of *typical set* inside the framework of statistical mechanics, thus strengthening the link between the two disciplines. Typicality is not a new word in statistical mechanics. It has been introduced in [6] for systems that are not at the equilibrium to mean that these systems, in their spontaneous evolution, *typically* tend to the maximum entropy. The difference with [6] is that (non) equilibrium is not our concern and that the typicality we refer to is the one that is technically established in information theory by the Shannon–McMillan theorem. Specifically, this paper shows that the Shannon–McMillan theorem *proves* that the microstates of the famous Boltzmann–Planck entropy formula are equiprobable. This result is surprising because, since the times of Boltzmann, the entire scientific community has postulated the equiprobability of microstates. Today, all the most influential textbooks of statistical mechanics and thermal physics unanimously say that microstates’ equiprobability is the central postulate of statistical mechanics; see for instance 4.2 of [7], chapter 6 of [8], 16.1 of [9], and 1.1 of [10]. In 3.4 of [11], the author not only postulates equiprobability but also expresses, in the passage that we hereafter quote, strong scepticism about the possibility of proving it.

*It is, unfortunately, impossible to prove with mathematical rigor that the principle of equal a priori probabilities applies to many-particle systems. Over the years, many people have attempted this proof, and all have failed. Not surprisingly, therefore, statistical mechanics was greeted with a great deal of scepticism when it was first proposed in the late 1800s. One of the its main proponents, Ludwig Boltzmann, became so discouraged by all of the criticism that he eventually committed suicide. Nowadays, statistical mechanics is completely accepted into the cannon of physics—quite simply because it works*.

The outline of the paper is as follows. In Section 2, we introduce our model of the thermodynamical system, where the only assumption that we make is that the system is made by a large number of non-interacting and identical particles. We say *our model* because it is slightly different from the commonly accepted one even if, in the thermodynamical limit of infinite number of particles, our model becomes compatible with the standard one. Section 3 shows that, with the further assumption of distinguishable particles, the set of microstates accessible to the thermodynamical system is the information-theoretic typical set of the Shannon–McMillan theorem. A crucial property of the typical set is that the probability distribution of its elements converges to uniformity; therefore, the identification between the typical set and the set of accessible microstates proves convergence to equiprobability of the accessible microstates. Finally, in Section 4 we recap the main points of the paper and draw conclusions.

## 2. Notation and System Model

Let uppercase calligraphic characters, e.g., X, denote discrete random variables; let the set {X} be the support set of the random variable, e.g.,

$$\left\{X\right\}=\left\{x_{0},x_{1},\cdots \right\};$$

and let |X| be the number of elements of the support set. The probability Pr(X=x) of the event X=x is denoted with the shorthand p(x),

Pr(X=x)=p(x),∀x∈{X}.

The probability distribution of X is the deterministic function p(·) of the random X with

$$\left\{p\left(X\right)\right\}=\left\{p\left(x_{0}\right),p\left(x_{1}\right),\cdots \right\}$$

and

Pr(p(X)=p(x))=p(x),∀x∈{X}.

Consider a thermodynamical system made by a fixed number *N* of non-interacting identical particles with one degree of freedom each. The thermodynamical system is modeled here by the random vector

$$E_{1}^{N}=\left(E_{1},E_{2},\cdots ,E_{N}\right)$$

of the energies of the *N* particles. We assume that energy is quantized and that the random entries of the random vector are independent (non-interacting), identically distributed (identical) (i.i.d.) random variables (particles); that is,

(1) $$p\left(E_{1}^{N}\right)=\prod_{i=1}^{N}p\left(E_{i}\right),$$

with

$$p\left(E_{1}\right)=p\left(E_{2}\right)=\cdots =p\left(E_{N}\right).$$

Since particles are i.i.d., one of them represents all. The random energy of the generic particle, which we call E, takes its values in the set {E} of the allowed energy levels

(2) $$\left\{E\right\}=\left\{\epsilon_{0},\epsilon_{1},\cdots \right\}.$$

The expected energy of the particle is

$$E=\sum_{\epsilon \in \{E\}}\epsilon p\left(\epsilon \right).$$

The total random energy of the system is

$$\sum_{i=1}^{N}E_{i}.$$

By the i.i.d. assumption and by the law of large numbers, when N→∞, the system’s random energy divided by the number of particles, that is, the random mean energy, tends to the deterministic expected energy:

(3) $$\lim_{N\to \infty }\frac{1}{N}\sum_{i=1}^{N}E_{i}=E.$$

Convergence of the limit can be in various senses. In this paper, we will focus our attention on the weak law of large numbers (Khinchin law); hence, we consider convergence of the limit (3) *in probability*, meaning that, for every η>0,

(4) $$\lim_{N\to \infty }Pr\left(\left|\frac{1}{N}\sum_{i=1}^{N}E_{i}-E\right|>\eta \right)=0.$$

The limit in (4) is interpreted by saying that, for every η, if *N* is sufficiently large, then the total energy of the system lies in a narrow interval ±Nη around NE with high probability.

A comment is in order about the approach pursued in this paper, which is based on the expected energy per particle, in comparison to the classical approach of thermal physics and statistical mechanics where the total system energy is considered. The assumption that the system’s total energy lies in a narrow interval is the basis of the classical *microcanonical ensemble* approach; see, for instance, chapters 1 and 2 of [10]. In the microcanonical ensemble approach, the system is assumed to have a large and fixed number of particles and to be closed. Since *N* is large and fixed, there is basically no difference between the expected energy per particle and the total energy divided by the number of particles. When the number of particles is fixed but not as large, the classical approach in statistical mechanics and thermal physics is that of the *canonical ensemble*; see, for instance, chapter 3 of [10]. In the canonical ensemble approach, the thermodynamical system is at the thermal equilibrium at a temperature *T* with a heat bath and interacts with it to maintain the equilibrium condition. One of the consequences of the interaction between the system and the heat bath is that the total energy of the system fluctuates; hence, it becomes *substantially* random. Due to the random nature of total system’s energy, in the canonical ensemble approach, it can no longer be used to characterize the system, which is characterized instead by the temperature of the heat bath. While the system’s total energy becomes substantially random and must be abandoned in favor of the temperature of the heat bath, the expected energy, which is deterministic, can be related in a deterministic way to the temperature of the heat bath by the appropriate energy–temperature relation. For instance, the high-temperature approximation for wholly kinetic energy is

$$E=\frac{kT}{2},$$

where *T* is in Kelvin degrees and *k* is Boltzmann’s constant. Hence, although in the following we will limit ourselves to N→∞, we see that the expected energy per particle can be used to characterize both the microcanonical and the canonical ensembles.

## 3. Equiprobability of Accessible Microstates

When we become aware of the result of a random experiment, in our case, the energy level of a particle, the *surprise* that we experience is the following deterministic function of the random result:

$$\begin{array}{c} -\log(p(E)), \end{array}$$

where log(x) indicates the logarithm of *x* and the base of the logarithm depends on the context. In information theory, the base is 2, while in physics, the base is Euler’s constant *e*. Being a function of a random variable, the surprise is itself a random variable. The expectation is Shannon’s entropy, which we call *H*:

(5) $$H=-\sum_{\epsilon \in \{E\}}p\left(\epsilon \right)\log\left(p\left(\epsilon \right)\right).$$

If distinguishable particles, or indexed particles, are considered, an outcome of $E_{1}^{N}$ is an *energy microstate*, or, simply, *microstate*. For instance, a microstate is

(6) $$E_{1}^{N}:\;E_{1}=\epsilon_{5},\;E_{2}=\epsilon_{31},\;\cdots ,\;E_{N}=\epsilon_{18},$$

meaning that the energy of the first particle is $\epsilon_{5}$, the energy of the second particle is $\epsilon_{31}$, ⋯, and the energy of the *N*-th particle is $\epsilon_{18}$. This definition of microstate matches the commonly accepted one; see, e.g., 1.1 of [10], where the microstates of the system are the independent solutions of the Schröedinger equation of the system whose eigenvalue is the total energy of the system. Due to the assumption of independency between particles, the system decouples into *N* independent equations whose individual solutions are the wave functions whose energy eigenvalues are the energy levels (2), the sum of the individual energy eigenvalues being equal to system’s total energy. For now, we settle for the definition of microstate; we will return soon on the concept of accessible microstate and on the energy constraint that the system must comply with.

When we become aware of the microstate visited by the system, the surprise that we experience is

$$\begin{array}{c} -\log(p\left(E_{1}^{N}\right)). \end{array}$$

The expectation of the above surprise is the Shannon entropy of the system; hence the Shannon entropy of the random vector $E_{1}^{N}$:

(7) $$-\sum_{\epsilon_{1}^{N}\in \left\{E_{1}^{N}\right\}}p\left(\epsilon_{1}^{N}\right)\log\left(p\left(\epsilon_{1}^{N}\right)\right).$$

In statistical mechanics, (7) is called *Gibbs entropy*. By the i.i.d. assumption (1), the entropy (7) is equal to *n* times the Shannon entropy of one particle:

$$-\sum_{\epsilon_{1}^{N}\in \left\{E_{1}^{N}\right\}}p\left(\epsilon_{1}^{N}\right)\log\left(p\left(\epsilon_{1}^{N}\right)\right)=NH;$$

see 2.6.6 of [4]. One immediately recognizes that

(8) $$\begin{array}{cc} \lim_{N\to \infty }-\frac{1}{N}\log\left(p\left(E_{1}^{N}\right)\right) & =\lim_{N\to \infty }-\frac{1}{N}\sum_{i=1}^{N}\log\left(p\left(E_{i}\right)\right) \end{array}$$

(9) $$\begin{array}{c} \;\;=H, \end{array}$$

where (8) is the assumption of independency between particles and (9) follows from the assumption of identically distributed random variables and from the law of large numbers. The equality between the leftmost term and the rightmost one in the above two equalities shows that, as N→∞, the distribution of the microstates that the system can visit converges to the uniform distribution, because *H* is a deterministic and fixed quantity, independent of the specific microstate $E_{1}^{N}$. The convergence of the limit (8) to (9) is true in various senses. Convergence in probability, that is

(10) $$\lim_{N\to \infty }Pr(|\frac{1}{N}\log\left(p\left(E_{1}^{N}\right)\right))+H|>\eta )=0,$$

is referred to as the Asymptotic Equipartition Property (AEP) in chapter 3 of [4], while many authors refer to (10) as to the Shannon–McMillan theorem (here it is the probability that is equally partitioned, not the energy; hence this AEP has nothing to do with the classical energy equipartition property). Almost everywhere convergence, that strengthens convergence in probability, is known as the Shannon–McMillan–Breiman theorem [12]. Exactly as it happens with total system’s energy, here, for sufficiently large but finite *N*, the surprise about the system’s microstate lies in a narrow range ±Nη around NH with high probability and, exactly as it happens with the expected energy per particle *E*, *H*, which is *per particle*, can be used to characterize the entropy both in the microcanonical and the in canonical ensemble approaches even if, in what follows, we will limit ourselves to N→∞.

### 3.1. Typical Set and Accessible Microstates

Starting from (10), we arrive at the definition of *typical set*, which is the subset $\left\{T_{1}^{N}\left(\eta \right)\right\}$ of $\left\{E_{1}^{N}\right\}$ made by the vectors $E_{1}^{N}$ whose probability, for any η and for sufficiently large *N*, is in the narrow range

(11) $$e^{-N(H+\eta )}\leq p\left(E_{1}^{N}\right)\leq e^{-N(H-\eta )}.$$

The properties of the typical set are that the number of its elements is in the narrow range

(12) $$e^{N(H+\eta )}\geq \left|T_{1}^{N}\left(\eta \right)\right|\geq \left(1-\eta \right)e^{N(H-\eta )}$$

and that the probability that the system visits one element of the typical set is

(13) $$Pr\left(E_{1}^{N}\right)\in \left\{T_{1}^{N}\left(\eta \right)\right\})>1-\eta ;$$

see again [4]. Formulas (11)–(13) show that with high probability, the system visits, or, in the language of statistical mechanics, *makes access to*, one among $|T_{1}^{N}\left(\eta \right)|$ microstates. In addition, the distribution of microstates converges to the uniform one as N→∞, because $|T_{1}^{N}\left(\eta \right)|$ is basically the inverse of $p(E_{1}^{N})$, which is constrained to lie in the narrow range (11). Note that convergence to equiprobability of accessible microstates is a *consequence* of the i.i.d. assumption and of N→∞, hence, in the end, of the law of large numbers, not a *postulate*.

The AEP divides the support set of energy microstates into two subsets: the typical set, which is the set of accessible microstates, and its complement, which is the set of microstates that the system cannot access. The division can be appreciated by observing that

$$\left|E\right|-e^{H}\geq 0,$$

with equality when the energy levels that the single particle can visit are equiprobable, hence

(14) $$|E_{1}^{N}{\left|\;=\;|E\right|}^{N}\geq e^{NH}.$$

Writing, with some abuse of mathematics, (12) as

$$|T_{1}^{N}\left(\eta \right)|\approx e^{NH},$$

we see that, when inequality (14) fits with equality, the size of the typical set is the entire support set made of ${\left|E\right|}^{N}$ elements, while, when $|E|>e^{H}$, the number of elements of the typical set is lower than ${\left|E\right|}^{N}$, leading to the division of the support set into the two subsets mentioned above. In this case, the system will visit with high probability the typical set and will visit with vanishingly small probability the complement of the typical set to the support set.

We now return to accessibility and the energy constraint to comment again on the key difference between our approach and the standard one. In the standard approach, for a microstate to be accessible, the energy eigenvalue of the wave function that solves the system’s Schröedinger equation of the system must be equal to the constraint of total energy that is imposed on the system, while here it is the probability distribution {p(E)} that must have a prescribed expected energy per particle. Compatibility between our approach and the standard one is guaranteed because, when N→∞, the energy eigenvalue of the system divided by the number of particles *is forced* by the law of large numbers to be equal to the expected energy of one particle; see (3).

### 3.2. Boltzmann–Planck Entropy

The equality between the Boltzmann–Planck entropy and *N* times the Shannon entropy of one particle is widely accepted in textbooks of statistical mechanics and thermal physics; see, e.g., Equation (18.14) of [9]. However, while in the standard approach, both the Boltzmann–Planck entropy and the Shannon entropy are purely deterministic quantities, in our approach, the Boltzmann–Planck entropy is the random quantity that we are will introduce in (18), that, divided by the number of particles, becomes equal to the deterministic Shannon entropy when the number of particles tends to infinity. We hereafter discuss the key passage, the passage where our model becomes compatible with the standard combinatorial approach to the evaluation of the number of accessible microstates.

Let N(ϵ) be the random number of particles that visit the generic energy level ϵ, with

(15) $$\sum_{\epsilon \in \{E\}}N\left(\epsilon \right)=N.$$

Compatibility of the accessible microstates with constraints imposed on the system will be guaranteed by imposing compatibility of the set

(16) $$\left\{N\left(\epsilon_{0}\right),N\left(\epsilon_{1}\right),\cdots \right\}$$

with the distribution {p(E)}. The random number of microstates is the multinomial coefficient

(17) $$W=\frac{N!}{\prod_{\epsilon \in \{E\}}N\left(\epsilon \right)!}$$

and the random Boltzmann–Planck entropy is

(18) S=klog(W)

Randomness of the set (16) and, as a consequence, of the number of microstates (17) and of the Boltzmann–Planck entropy (18) marks the difference between our approach and the standard, deterministic one. Exactly as in the case of the energy, once again the law of large numbers bridges the gap between randomness and determinism, thus making our *random* approach compatible with the *deterministic* standard approach, hence the key passage is

(19) $$\lim_{N\to \infty }\frac{1}{N}\log\left(W\right)=H.$$

Specifically, step by step, one has

$$\begin{array}{cc} & \lim_{N\to \infty }\frac{1}{kN}S=\lim_{N\to \infty }\frac{1}{N}\log\left(W\right)\\ & =\lim_{N\to \infty }\frac{1}{N}(\log\left(N!\right)-\sum_{\epsilon \in \{E\}}\log\left(N\left(\epsilon \right)!\right)) \end{array}$$

(20) $$\begin{array}{c} \;\;\;\;\;=\lim_{N\to \infty }\frac{1}{N}(N\log(\frac{N}{e})-\sum_{\epsilon \in \{E\}}N\left(\epsilon \right)\log(\frac{N(\epsilon )}{e})) \end{array}$$

(21) $$\begin{array}{c} \;\;\;=\lim_{N\to \infty }\frac{1}{N}(N\log\left(N\right)-\sum_{\epsilon \in \{E\}}N\left(\epsilon \right)\log\left(N\left(\epsilon \right)\right)) \end{array}$$

(22) $$\begin{array}{c} \;\;\;=\lim_{N\to \infty }\sum_{\epsilon \in \{E\}}\frac{N(\epsilon )}{N}(\log(N)-\log(N(\epsilon )))\\ \;\;\;=\lim_{N\to \infty }-\sum_{\epsilon \in \{E\}}\frac{N(\epsilon )}{N}\log(\frac{N(\epsilon )}{N}) \end{array}$$

(23) $$\begin{array}{l} =-\sum_{\epsilon \in \{E\}}p\left(\epsilon \right)\log\left(p\left(\epsilon \right)\right) \end{array}$$

$$\begin{array}{ll} & =H, \end{array}$$

where in (21) and (22) we use (15), (23) is the law of large numbers and (20) is Stirling’s formula that, in the big O notation, is

$$\log\left(N!\right)=N\log(\frac{N}{e})+O\left(\log\left(N\right)\right),$$

and we put in (20)

$$\lim_{N\to \infty }\frac{O(\log(N))}{N}=\lim_{N\to \infty }\frac{O(\log(N(\epsilon )))}{N}=0,\;\;\forall \;\epsilon \in \left\{E\right\}.$$

The law of large numbers is also invoked in [13] in a passage that is basically the same as our (23) to show the equivalence of the phase space formulation of Gibbs and Boltzmann–Planck entropies.

From (12) and (19) we conclude that, for every small η>0,

(24) $$\lim_{N\to \infty }\frac{1}{N}\log\left(W\right)=\lim_{N\to \infty }\frac{1}{N}\log(|T_{1}^{N}\left(\eta \right)\left|\right).$$

Therefore, not only is our definition of energy microstates exemplified in (6) the commonly accepted one, but also the logarithm of the number of accessible microstates that we obtain by our approach is, for N→∞, equal to the logarithm of the standard combinatorial definition (17) of accessible microstates.

## 4. Conclusions

This paper has demonstrated that there is no need to *assume* or *postulate* the equiprobability of accessible microstates, because convergence of microstates’ distribution to uniformity is a consequence of the large number of particles, of the i.i.d. assumption and of the assumption of distinguishable particles. Specifically, Equations (11)–(13) show that convergence to equiprobability is a consequence of the AEP, and Equation (24) shows that the microstates we are dealing with are the microstates that are counted by the standard combinatorial approach.

It is worth observing that equiprobability has nothing to do with the thermal equilibrium and hence with entropy maximization. Actually, the only conditions for convergence to equiprobability are the assumptions that we made, which do not include thermal equilibrium and/or entropy maximization; therefore, the distribution converges to the uniform one also if the system is not at the thermal equilibrium. What can change between a system at the equilibrium and a system that is not at the equilibrium when both are subject to the same constraints is the probability distribution of energy levels and, with it, Shannon’s entropy and, with it again, the number of accessible microstates, which, however, tend to equiprobability. Since the accessible microstates also remain virtually equiprobable in systems that are not at the equilibrium, the entropy of these systems is still given by the Boltzmann–Planck entropy formula, provided that the number of accessible microstates is conveniently expressed through the Shannon entropy of the specific (non-equilibrium) probability distribution of the energy of the individual particle by (5), (12).

When *N* is not large, e.g., N=1, microstates are in general not equiprobable, independently of the entropy of the system.

To summarize, the main result of this paper is the following:

- For large *N* and i.i.d. particles, the accessible microstates are always virtually equiprobable, and also for systems that are not at the thermal equilibrium;
- For small *N*, the accessible microstates are never equiprobable, and also for systems that are at the thermal equilibrium with a heat bath.

The important case of indistinguishable particles is not treated in the paper and is left to future research.

## Data Availability

Not applicable.
